# Perceived and Observed Treatment Gains Following PEERS: A Preliminary Study with Latinx Adolescents with ASD

**DOI:** 10.1007/s10803-022-05463-9

**Published:** 2022-02-14

**Authors:** Elina Veytsman, Elizabeth Baker, Ann Marie Martin, Tricia Choy, Jan Blacher, Katherine Stavropoulos

**Affiliations:** Graduate School of Education, University of California, 1207 Sproul Hall, Riverside, CA 92521 USA

**Keywords:** Program for the Education and Enrichment of Relational Skills, Social skills, Adolescents, Latinx, Contextual Assessment of Social Skills

## Abstract

The Program for the Education and Enrichment of Relational Skills (PEERS) social skills intervention has demonstrated effectiveness for adolescents with autism spectrum disorder (ASD). However, studies have been limited by a lack of objective outcome measures and an underrepresentation of Latinx families. This pilot study extends the PEERS literature by utilizing an observational measure of conversational skills (Contextual Assessment of Social Skills; CASS) with a diverse sample of 13 adolescents with ASD (with parent groups conducted in English and Spanish simultaneously) and a control group of 11 neurotypical adolescents. Consistent with previous research, adolescents with ASD and their parents perceived improvements in social functioning following intervention, which were maintained four months later and corroborated by improvements in conversational skills.

## Introduction

Adolescents with autism spectrum disorder (ASD) experience challenges with social interaction and communication, affecting their ability to develop and maintain friendships (Reichow & Volkmar, 2010). One challenge during adolescence is the increased emphasis on peer relationships, and the expected decreased reliance on parents. For adolescents with ASD, this may be a particularly difficult period, marked by negative social outcomes, such as fewer friends, lack of social support, increased peer rejection, and limited social engagement (Shattuck et al., 2011).

The complexity of conversational skills, in particular, is an area of difficulty for autistic adolescents,^1^ in part due to underlying deficits in social communication, social awareness, social motivation, and social cognition (Carter et al., 2005; Chevallier et al., 2012). Various theories have been proposed to understand the nature of these difficulties, including the weak central coherence theory (Happe & Frith, 2006) and the systemized processing bias (Baron-Cohen et al., 2003). The weak central coherence theory poses that autistic individuals have a cognitive perceptual deficit in global information processing, or “seeing the big picture”, which may relate to difficulties with integrating social information. The systemized processing bias suggests that rather than having weak overall global processing, autistic individuals have a higher ability to predict the behavior of a system (systemizing) than the behavior of a person (empathizing), which in effect, leads to social difficulties and withdrawal. Correspondingly, adolescents with ASD are typically less involved in social activities and thus have fewer opportunities to practice social interactions in comparison to neurotypical youth, limiting their conversational opportunities (Chevallier et al., 2012; Shattuck et al., 2011). These conversational challenges and reduced social interactions make developing and maintaining friendships difficult for adolescents with ASD (Laugeson & Frankel, 2010).

Despite the social challenges experienced by youth on the spectrum, there is ample evidence suggesting a desire for social involvement and the development of social relationships. For example, Church et al. (2000) found that autistic adolescents in middle school with average to above average cognitive abilities expressed some interest in interacting with their peers. It is not unusual for these adolescents to report concerns over making friends or not having any friends (Locke et al., 2010). Taken together, the evidence suggests that adolescents with ASD report poorer quality friendships, greater loneliness, and greater social anxiety than their neurotypical peers (Bauminger & Kasari, 2000).

Adolescents with ASD with average to high cognitive abilities may initiate more social interactions compared to their peers with lower cognitive abilities; however, their higher cognitive awareness and recognition of their own lack of friends may actually increase their likelihood of loneliness (Bauminger et al., 2003), peer rejection (Church et al., 2000), and bullying (Zeedyk et al., 2014). Given the reported challenges with developing and maintaining friendships, social skills have been a primary target for intervention, especially among adolescents with ASD with cognitive abilities in the average range. With an increase in the prevalence of ASD in the last few decades, there has also been an increase in research evaluating the effectiveness of group-based social skills interventions (GSSIs; Gates et al., 2017). One of the most well-studied GSSIs for adolescents with ASD is the Program for the Education and Enrichment of Relational Skills (PEERS; Laugeson et al., 2009, 2012). However, research on GSSIs, including PEERS, has been limited by methodological issues, such as limited objective outcome measures and a lack of focus on skill generalization (Wolstencroft et al., 2018; Zheng et al., 2021).

Though the PEERS intervention has a strong literature base relative to other social skills interventions for adolescents with ASD, most of the reported treatment gains have come from the participants themselves. Informants have their own biases, attributions, and expectations that clearly influence their responses (Stratis & Lecavalier, 2015). Self-report measures are commonly used in the assessment of youth with social challenges, but numerous research studies have suggested that self-report of adolescents with ASD should be interpreted with caution (Stratis & Lecavalier, 2015). One such study found that children and adolescents with ASD reported greater levels of social skills and social competence, compared to parent reports of the same constructs (Knott et al., 2006). The use of multiple informants in the assessment of psychological functioning is considered a “gold standard” in the assessment of psychopathology in children and adolescents (e.g., Mash & Hunsley, 2005). However, due to their inherent bias, questionnaire measures should be combined with other more objective measures of treatment outcomes, such as behavioral observations, cognitive or neuropsychological measures, and sociometric tasks (Gates et al., 2017; Kaat & Lecavalier, 2014). Observations of behavioral change by blind raters may be one of the most objective measures of treatment outcome, but they are used less frequently than questionnaire measures (Kaat & Lecavalier, 2014). For example, only two of the eight RCTs conducted on the PEERS for Adolescents intervention have utilized observational measures (Rabin et al., 2018; Yoo et al., 2014).

An observational measure that has shown promise as an ecologically valid treatment outcome measure following PEERS is the Contextual Assessment of Social Skills (CASS; Dolan et al., 2016; Rabin et al., 2018; Ratto et al., 2011; White et al., 2015). The CASS is a semi-structured live role-play measure of conversational skills consisting of a three-minute filmed conversation between the adolescent and an unfamiliar peer (i.e., a research confederate). Simmons et al. (2020) evaluated the utility and validity of the CASS as a measure of social cognition and social behavior for adolescents with ASD, finding the CASS to have strong internal and external validity. Results of this study suggest that the CASS should be used as part of a multimethod battery for assessing outcomes of clinical interventions in individuals with ASD.

In addition to a lack of objective forms of assessment, there is a lack of reported diversity in GSSI studies. Though cross-cultural replication studies with PEERS have been conducted with Asian and European samples (e.g., Rabin et al., 2018; Shum et al., 2018; Yamada et al., 2020; Yoo et al., 2014), Latinx families, especially those who are socioeconomically disadvantaged, have been consistently underrepresented in ASD intervention research (Bernal & Domenech Rodríguez, 2009; Pickard et al., 2019; Ratto et al., 2017). Previous research on adapting ASD interventions for Latinx samples indicates that culturally sensitive adaptations are necessary for successful intervention implementation (Chlebowski et al., 2020; Huey & Polo, 2008; Ratto et al., 2017). Thus, one objective of this study as a whole was to examine whether Latinx families achieve similar benefits after participation in PEERS. Finally, though waitlist and no-treatment control groups have been employed in many PEERS studies, no studies have utilized a typically developing, no-treatment control group to evaluate whether improvements in social competence among ASD participants are clinically meaningful and based on the intervention itself (Zheng et al., 2021).

While the focus of this study is evaluating a social skills intervention for autistic teens, it is important to acknowledge that the neurodiversity paradigm has shifted the focus of autism intervention research away from the neurodivergent individual and towards society (e.g., Jurgens, 2020). In line with the neurodiversity paradigm, the social model of autism rejects the view that autism is a disability and instead emphasizes a strengths-based perspective in which interventions are focused on improving autistic individuals’ capabilities and well-being, rather than correcting their perceived ‘deficits’. It should therefore be noted that many of the measures of social functioning used in this study were created based on the assumption that various skills should be judged with neurotypical individuals as the ‘standard’ (e.g., measuring social skills using the framework that neurotypical social behavior is ‘correct’ and that autistic individuals can ‘improve’ their social skills). Despite these inherent limitations, the measures used in the current study have been validated across many studies and continue to be widely used to assess social functioning in autistic individuals. Thus, when we refer to “improvements” in social functioning or “higher” or “lower” social skills, we are doing so in the context of the norms of these measures.

## Objectives

This pilot study aimed to: (1) Replicate previous PEERS studies by examining self- and parent- reported treatment gains at pre- and post-intervention and at a four-month follow-up; and (2) extend previous findings by including (a) a diverse sample of predominantly Latinx families, (b) an objective observational measure of treatment outcome (i.e., the CASS), and (c) a no-treatment control group of neurotypical (TD) adolescents (i.e., adolescents not participating in the intervention) to assess whether improvements were due to treatment and can be considered clinically meaningful. The following research questions were addressed:

1. Did the PEERS intervention produce improvements in social skills as determined by teen and parent report? Were these improvements statistically significant as well as clinically meaningful (i.e., did the level of social functioning of teens with ASD improve sufficiently to be similar to normative scores from TD teens)?
2. Were improvements in social functioning corroborated by improvements in observed conversational skills as rated by blind independent raters?

## Methods

### Recruitment and Eligibility

Families were recruited from local school districts, community organizations, parent advocacy groups, and flyers posted in the community. At initial recruitment, adolescent eligibility criteria included (a) age range from 11 to 18 years and currently in middle school or high school, (b) ability to speak and understand English, and (c) willingness to participate. Exclusionary criteria for youth included (a) a history of major mental illness (e.g., psychosis), (b) hearing, visual, intellectual, or physical disabilities, (c) current problems with severely aggressive or oppositional behaviors, (d) history of seizure disorder, and/or (e) other physical or neurological illnesses that would inhibit participation in treatment. Additionally, exclusionary criteria for the TD group included immediate family history of ASD or developmental disabilities. Finally, criteria for parent participants included (a) ability to understand and speak either English or Spanish, and (b) commitment to consistently attend sessions and participate as the teen’s social coach. This study was approved by the university’s Institutional Review Board. Parents provided consent and adolescents provided assent to participate.

## Participants

A total of 17 adolescents with ASD were enrolled in the study; however, 4 dropped out of the intervention due to difficulty with transportation, psychiatric hospitalization, or the adolescent no longer wanting to attend intervention sessions. It is noteworthy that the attrition rate in this study was slightly higher than that of previous PEERS studies (i.e., 24% compared to 8–18% in other studies) (e.g., Rabin et al., 2018; Schohl et al., 2014; Yoo et al., 2014). Thus, the final sample consisted of 13 adolescents with ASD (ten males; 69% Latinx), ages 11–17 (*M* = 14.17, *SD* = 2.09), and their parents, recruited at two time-points. The first cohort consisted of seven adolescents, and the second cohort consisted of six adolescents. In addition, 11 control TD participants (nine males; 55% Latinx), ages 11–17 (*M* = 13.1, *SD* = 1.3) were recruited to assess the stability of scores across time and to have a point of comparison for treatment gains among ASD participants; these adolescents did not participate in treatment. See Table 1 for detailed information on participant demographics.

**Table 1:** Participant demographic variables for ASD and TD groups

	Group		*p*
	ASD (*n* = 13)	TD (*n* = 11)	
*Adolescent variables*			
Mean Age (SD)	14.2 (2.1)	13.1 (1.3)	Ns
Mean Grade (SD)	8.6 (2.1)	7.7 (1.2)	Ns
WASI-2 FSIQ (SD)	99.5 (15.6)	112.7 (11.1)	0.029
Sex (% male)	76.9	81.8	Ns
School type (%)	53.8 middle school, 46.2 high school	45.5 middle school, 36.4 high school, 18.2 elementary	Ns
% with co-occurring diagnosis	38.5 ADHD 23.0 other	N/A	N/A
School setting (% mainstreamed)	60.2	N/A	N/A
Services (% receiving)	53.8 speech, 23.1 mental health, 23.9 other	N/A	N/A
Stimulant medication (% currently taking)	15.4	N/A	N/A
*Parent variables*			
Parent 1 education level (% with Bachelor’s degree or higher)	23.1	90.9	0.004
Parent 2 education level^a^ (% with Bachelor’s degree or higher)	20.0	83.3	0.010
Parent 1 ethnicity (%)	69.2 Latinx, 30.8 Caucasian	54.5 Latinx, 36.4 Caucasian, 9.1 Asian	Ns
Parent 2 ethnicity^a^ (%)	63.6 Latinx, 27.3 Caucasian, 9.1 Black	50.0 Caucasian, 33.3 Latinx, 16.7 Asian	Ns
Parent 1 country of birth (%)	69.2 U.S., 30.8 Mexico	70.0 U.S., 20.0 Mexico, 10.0 Thailand	Ns
Parent 2 country of birth^a^ (%)	63.6 U.S., 36.4 Mexico	57.1 U.S., 28.6 Mexico, 14.3 Thailand	Ns

^a^*n* = 11 for ASD group and *n* = 6 for TD group.

## Procedure

For adolescents participating in PEERS, eligibility was initially assessed during a phone screening interview with the parent using the Phone Screening Script (Laugeson & Frankel, 2010). Adolescents’ motivation to participate was assessed during a brief phone call with the adolescent, and again during an intake appointment using the Teen Mental Status Checklist from the manual. Prior to participating in treatment, all families came in individually to the university autism center for an intake appointment. During this appointment, informed parental consent and adolescent assent were obtained, and the adolescents were administered the Autism Diagnostic Observation Schedule, 2nd edition (ADOS-2; Lord et al., 2012) to confirm autism eligibility, and the Wechsler Abbreviated Scale of Intelligence, 2nd edition (WASI-II; Wechsler, 2011) to determine that their IQ was 70 or above. Adolescents and parents completed various questionnaires, including a demographic form and measures of adolescent social functioning, parent acculturation and language, and parent and family impact. Adolescents also participated in a three-minute conversational interaction with an unfamiliar peer (i.e., the CASS).

Within two weeks following the 16-week treatment, adolescents and parents came in for a post-appointment and completed all of the same measures from the intake appointment, excluding the diagnostic and cognitive assessments, demographic questionnaire, and Teen Mental Status Checklist. Four months after the completion of the intervention, families came in for a follow-up appointment, which was identical to the post-appointment, in order to assess maintenance of treatment gains. The second cohort of adolescents and parents completed their follow-up measures online via Qualtrics due to in-person COVID-19 restrictions. With the exception of the CASS, TD participants completed the same measures in person at three timepoints, each four months apart, to assess the stability of scores over time; for three TD participants, follow-up measures were completed via Qualtrics due to COVID-19 restrictions. To increase retention rates, families were each compensated $110, spread out throughout the pre, post, and follow-up appointments.

## Treatment

The PEERS Curriculum for School-Based Professionals (Laugeson, 2014) comprised the intervention. It was administered in a 16-week format, and was used concurrently with the original PEERS Treatment Manual (Laugeson & Frankel, 2010) for the parent portion of the intervention. Adolescents and parents attended 90-minute concurrent but separate sessions. Treatment was conducted by two PEERS Certified Providers, and all procedures were overseen by a licensed psychologist. Behavioral coaches who were undergraduate or graduate students assisted with role-play demonstrations, behavior management, attendance and homework tracking, and tracking treatment fidelity.

For teens, treatment sessions used didactic instruction in a small group format, which included role-play demonstrations, behavioral rehearsal activities with reinforcement and corrective feedback, and weekly homework assignments related to social engagement (Ellingsen et al., 2017). To promote generalization of the skills outside of the clinic setting, parents were taught how to become social coaches for their teens by using key words taught by the program when providing feedback or practicing skills at home, and identifying appropriate extracurricular activities that can serve as a source of friends for their teens (Ellingsen et al., 2017; Laugeson et al., 2009, 2012). Teens received points for completing homework assignments and for participation during the didactic lesson and behavioral rehearsal exercises. See Table 2 for detailed information regarding program participation and treatment compliance.

**Table 2:** Program participation and treatment compliance for ASD participants (*n* = 13)

Variables	Mean	SD	Range
Teen attendance	14.9	1.4	12—16
Parent attendance	15.0	1.0	13—16
Teen-reported homework completion	66.3	12.8	41.2—84.3
Parent-reported homework completion	66.0	13.8	41.2—89.2
Teen total points earned	256.3	91.4	165—469

*Note.* Attendance = # of sessions attended out of 16. Homework completion = % completed assignments.

Topics of instruction included using appropriate conversational skills; choosing appropriate friends; using electronic communication appropriately and safely; using humor appropriately; initiating, joining, and exiting conversations with peers; organizing successful get-togethers; being a good sport when playing games/sports with peers; handling arguments and disagreements; handling rejection, teasing, bullying, rumors/gossip and cyber bullying; and changing a bad reputation (Laugeson, 2014). Treatment fidelity was assessed using a checklist. Behavioral coaches were responsible for ensuring that the group leader covered each component of the intervention in the treatment manual. 100% treatment fidelity was reported in both the adolescent and parent groups.

### Spanish Translation

In an effort to adapt the PEERS intervention for Latinx families, materials provided to families were professionally translated into Spanish prior to the start of the program, including the parent handouts and homework assignment sheets, the program welcome letter, the planned absence sheet, and the graduation flyer. The adolescent groups were conducted in English, and the parent groups were conducted simultaneously in English and Spanish by a bilingual group leader. As many of the parents recruited for this study were bilingual, a combination of English and Spanish in the parent group was determined to be the most culturally sensitive and inclusive format, with additional supports as needed to maintain participation comfort (e.g., a one-on-one translator for parents with lower levels of English comprehension).

## Measures

### Descriptive Measures

The following measures were administered at the initial screening appointment in order to confirm eligibility for participation in treatment.

#### Autism Diagnostic Observation Schedule, 2nd edition (ADOS-2; Lord et al., 2012)

The ADOS-2 is a play- and interview-based semi-structured standardized assessment of communication, social interaction, and play for individuals with suspected diagnoses of ASD. In the current study, Modules 3 and 4 of the ADOS were administered (to ASD participants only) by examiners trained to research-level reliability, in order to confirm previous diagnoses of ASD.

#### Wechsler Abbreviated Scale of Intelligence (WASI-II; Wechsler, 2011)

The WASI-II is a brief and reliable measure used to assess cognitive ability in individuals ages 6 to 90. For the current study, two subtests, Vocabulary and Matrix Reasoning, were combined to form the Full-Scale IQ-2 (FSIQ-2).

### Outcome Measures

The following measures were administered to both the ASD and TD groups at pre- and post- treatment and at a four-month follow-up.

#### Social Skills Improvement System (SSIS; Gresham & Elliott, 2007)

The SSIS is a standardized 79-item parent-report measure of social and behavioral functioning for children ages 3–18. Parents are asked to indicate how often their child displays a particular behavior (e.g., “introduces him/herself to others”), by rating items on a 4-point Likert scale as “never”, “seldom”, “often”, or “almost always.” The Social Skills and Problem Behaviors standard scores were used to provide summary ratings of treatment-related changes in social skills and problem behaviors.

#### Social Responsiveness Scale, 2nd Edition (SRS-2; Constantino & Gruber, 2012)

The SRS-2 is a standardized 65-item parent-report rating scale used to assess the severity of autism symptoms as they occur in natural settings for children ages 4–18. Parents indicate how often their child has displayed social behaviors characteristic of ASD (e.g., “has difficulty relating to peers”) in the past six months by rating items on a 4-point Likert scale from “not true” to “always true.” The total T-score was used to reflect overall social responsiveness.

#### Quality of Socialization Questionnaire (QSQ; Laugeson et al., 2009)

The QSQ is a 12-item questionnaire administered separately to parents and adolescents to assess the frequency of hosted and invited get-togethers in the previous month, the number of friends involved, and the level of conflict during these get-togethers. Consistent with previous studies evaluating the effects of social skills training (e.g., Laugeson et al., 2012), only the two items assessing the number of hosted and invited get-togethers were used in this study.

#### Test of Adolescent Social Skills Knowledge—Revised (TASSK-R; Laugeson & Frankel, 2010)

The TASSK-R is a 30-item criterion-referenced self-report measure that assesses an adolescent’s knowledge of the social skills taught in the PEERS intervention. Items include sentence stems in which adolescents choose the best option from two possible choices, based on the PEERS didactic lessons. The total score was used to reflect PEERS-specific social skills knowledge; higher scores indicate greater social skills knowledge.

#### Social Interaction Anxiety Scale (SIAS; Mattick & Clarke, 1998)

The SIAS is a 20-item self-report measure that assesses an adolescent’s fears around social interaction (e.g., being boring, sounding stupid, being ignored). All items are rated on a 5-point scale based on the degree to which respondents feel that the given statement is characteristic of them. The total score was used to reflect social interaction anxiety; higher scores indicate greater anxiety.

#### Loneliness and Social Dissatisfaction Questionnaire (LSDQ; Asher et al., 1984)

The LSDQ is a 24-item, standardized self-report measure of loneliness and social inadequacy. 16 items are focused on feelings of loneliness and social dissatisfaction, and the other 8 are filler items. Items are on a Likert scale from 1 (not true at all) to 5 (always true). Scores range from 16 (high loneliness) to 80 (low loneliness), with greater scores indicating greater social satisfaction.

#### Piers-Harris Self-Concept Scale-Second Edition (PH-2; Piers, 1984)

The PH-2 is a 60-item self-report measure that assesses teens’ self-esteem and self-concept. Teens are asked to circle a “yes” or “no” response for descriptive statements. The total score reflects overall self-concept, while subscale scores provide more detailed interpretation about specific dimensions (e.g., Happiness and Satisfaction; Popularity). Higher scores indicate more positive self-concept.

#### Friendship Quality Scale (FQS; Bukowski et al., 1994)

The FQS is a 23-item self-report measure that examines the quality of the teen’s best friendships. Items are on a Likert scale from 1 (not true) to 5 (really true). Adolescents are instructed to write the name of their best friend and answer the items with this friendship in mind; higher total scores indicate better quality best friendships.

#### Contextual Assessment of Social Skills (CASS; Ratto et al., 2011)

The CASS, an observational measure of conversational skills, was only administered to adolescents with ASD. Adolescent participants are asked to have a three-minute conversation with an unfamiliar peer (i.e., a research confederate), who was not involved in the current intervention. The participant and confederate are told to “act as if you have recently joined a new club or social group”; the examiner then leaves the two in the room together to talk. The filmed interaction is then coded for nine items, including two frequency counts (number of questions asked, number of initiated topic changes), and seven 1–7 Likert scale items (vocal expressiveness, gestures, positive affect, kinesic arousal (i.e., body movement/fidgeting), social anxiety, involvement in the conversation, and quality of rapport). The last two Likert scale items (conversational involvement and rapport) represent global ratings of conversational skills, and can be considered the most salient representation of social competence of the nine items. For the Likert scale items, a specific qualitative description accompanies each score; higher scores indicate better conversational skills (for specific codes, see Ratto et al., 2011).

The original CASS paradigm included an “interested” and a “bored” condition, in order to measure an individual’s ability to adapt their social behavior to the social context (Ratto et al., 2011). The current study used only the “interested” condition, as previous research on the CASS suggested that this condition would be the most likely to reflect changes following PEERS (Dolan et al., 2016; Rabin et al., 2018; White et al., 2015). Three females and five males served as CASS confederates; five were undergraduate students, and two were graduate students. Confederates received approximately one hour of training, using the CASS script developed by Ratto et al. (2011). Confederates were also taught to appropriately time pauses (e.g., allowing five seconds before speaking) and to use prompts to maintain the conversation, while minimizing social initiation, to allow the participants ample opportunities to initiate. Following each CASS session, confederates watched their filmed interaction and received feedback on their performance in order to improve standardization for future sessions.

Prior to the start of the study, the developer of the CASS conducted an off-site training with the author and research team on the measure’s development, administration, and scoring. Four undergraduate coders (none of whom served as confederates) were then trained to reliability, by rating four training videos provided by the authors (Ratto et al., 2011). The coders were required to achieve 80% agreement with Ratto et al.’s “gold” codes in order to become reliable; inter-rater agreement occurred when raters were within one point of each other. Coders had high reliability with the authors’ “gold” codes on the training videos (0.83–0.86). For the current study, inter-rater reliability was established by double coding 100% of the videos; two separate coding teams coded the videos for the two cohorts. Coders were blind to treatment status (pre, post, or follow-up). Inter-rater reliability for the current study’s videos was 0.74 (cohort 1) and 0.88 (cohort 2). Note: Due to COVID-19, the CASS was not administered at the four-month follow-up for the second cohort of six teens. Thus, only pre-post analyses were conducted with the CASS to maintain consistency between the two cohorts.

### Data Analysis

All analyses were conducted using SPSS 24.0 (SPSS, Inc., 2018). To assess change in adolescent and parent perceptions of social functioning across time, two (group) by three (time) repeated measures analyses of variance (ANOVAs) were conducted for each questionnaire measure. Bonferroni posthoc tests were conducted to examine differences between groups and between timepoints for both ASD and TD participants. Effect size estimates were calculated in SPSS using the partial eta squared statistic. For measures demonstrating improved average scores for ASD participants but a lack of statistical significance in the two (group) by three (time) ANOVA, a within-subjects repeated measures ANOVA was conducted to examine changes in the ASD group from pre- to post- treatment. This was to account for the potential unintended impact of the COVID-19 pandemic and resulting stay-at-home order on follow-up scores (i.e., due to reduced opportunities for social engagement, and possible challenging behaviors accompanying the transition to distance learning).

To examine changes in conversational skills on the CASS from pre- to post- treatment, descriptive statistics were analyzed to assess changes in means on the nine codes of the CASS. Similar to the White and Rabin et al. studies (2015 and 2018, respectively), the CASS total score was computed by adding each participant’s scores for question asking, topic changes, conversational involvement, and quality of rapport. Paired samples T-tests were conducted for each CASS code to assess change from pre- to post-treatment. Next, mean difference scores were computed for the two global CASS items (overall involvement and rapport) and the CASS total score to represent change in conversational skills following treatment. To assess whether improvements in CASS scores related to improvements on rating scales of social functioning, bivariate Pearson correlations were conducted between the mean difference scores of the global CASS items and CASS total score and the questionnaire measures that showed significant improvements in the repeated measures ANOVAs.

## Results

Results addressing impact of the PEERS intervention on social functioning as reported by the adolescent participants will be presented first, followed by the analyses examining parent reports and observed conversational skills.

### Adolescent Self-Report Measures

To evaluate the impact of the PEERS intervention on adolescent social skills knowledge, the total TASSK score was examined; a significant group by time interaction was observed (*F*(2,42) = 38.72, *p* < 0.001, *η*_*p*_^2^ = 0.65). A Bonferroni post-hoc test showed that social knowledge improved significantly from pre- to post- treatment for ASD participants (*p* < 0.001), and was stable from post-treatment to follow up (*p* = 0.54). TD teens not participating in treatment had stable scores on the TASSK across time-points (*p*’s > 0.05). At pre-treatment, ASD and TD participants had similar levels of social knowledge on the TASSK (*p* = 0.88). However, at post-treatment and follow-up, ASD participants had greater PEERS-specific social knowledge than did TD participants (*p*’s < 0.001).

In terms of adolescent-reported social engagement (QSQ), the group by time interaction was not significant for hosted get-togethers (*F*(2,42) = 0.02, *p* = 0.98, $\eta_p^{2}$ = 0.001) or invited get-togethers (*F*(2,42) = 0.20, *p* = 0.82, $\eta_p^{2}$ = 0.009). However, to account for the potential effects of the COVID-19 pandemic on get-togethers, a two-way within-subjects repeated measures ANOVA was conducted for the ASD group, demonstrating that teens with ASD reported hosting significantly more get-togethers from pre- to post- intervention (*F*(1,12) = 5.82, *p* = 0.03, $\eta_p^{2}$ = 0.33). In contrast, there were no significant changes in the frequency of invited get-togethers from pre- to post- treatment for adolescents with ASD (*p* > 0.05). TD adolescents reported a stable frequency of QSQ hosted and invited get-togethers across time (all *p*’s > 0.05). Notably, TD participants reported significantly more hosted get-togethers than ASD participants at pre-treatment (*p* = 0.001) and follow-up (*p* = 0.04); however, between-group differences were not significant at post-treatment (*p* = 0.28). Similarly, between-group differences in invited get-togethers were significant at pre-treatment (*p* = 0.04), but were not significant at post-treatment or follow-up (*p*’s > 0.05). Note that follow-up data were collected during the COVID-19 stay-at-home order in California, during which get-togethers were highly discouraged.

Since follow-up data on anxiety were not collected from TD participants, a three-way within-group repeated measures ANOVA was conducted for the ASD group only. There was a marginal effect of time on SIAS social anxiety scores for adolescents with ASD (*F*(2,24) = 3.31, *p* = 0.08, $\eta_p^{2}$ = 0.22). A Bonferroni post-hoc test also demonstrated marginal improvements in social anxiety from pre- to post- intervention for ASD participants (*p* = 0.05), which were maintained at follow-up (*p* > 0.05). TD participants had stable SIAS scores from pre- to post-treatment (*p* = 0.62). There were no significant between-group differences in social anxiety at pre- or post- intervention (*p*’s > 0.05).

For both ASD and TD participants, loneliness scores on the LSDQ were stable across time. Participants with ASD reported significantly more loneliness on the LSDQ than TD participants across time (all *p*’s < 0.05). Self-concept scores on the Piers-Harris were also stable across time and no significant effects were observed. Friendship quality scores on the FQS were also stable across time for both groups. Notably, between-group differences in friendship quality were significant at pre-treatment (*p* = 0.05), but were not significant at post-treatment or follow-up (*p*’s > 0.05).

### Parent-Report Measures

The group by time interaction was not significant for parent-reported social skills on the SSIS. However, for ASD participants, a three-way within subjects repeated measures ANOVA demonstrated a significant main effect of time on SSIS social skills (*F*(2, 24) = 12.83, *p* < 0.001, $\eta_p^{2}$ = 0.52). Bonferroni post-hoc tests showed significant improvements on the SSIS from pre- to post- treatment (*p* < 0.01), which remained stable at follow up (*p* > 0.05). TD participants had stable SSIS social skills scores across time (*p*’s > 0.05), and demonstrated significantly higher overall social skills compared to ASD participants at all time points (all *p*’s < 0.05). In terms of problem behaviors on the SSIS, the group by time interaction was not significant. Between-group differences were found at all time points, such that parents of ASD participants reported significantly greater problem behaviors than parents of TD participants (all *p*’s < 0.05). However, there was a marginal effect of time on problem behaviors for ASD participants in the within-subjects three-way repeated measures ANOVA (*F*(2,24) = 2.79, *p* = 0.08, $\eta_p^{2}$ = 0.20). Post-hoc comparisons did not show significant effects; however, a two-way (pre- and post-intervention) ANOVA indicated significant improvements in problem behaviors from pre- to post- treatment (*F*(1,12) = 5.09, *p* = 0.04) for ASD participants.

There was a significant group by time interaction on parent-reported social responsiveness, as indicated by the SRS total score (*F*(2, 44) = 4.22, *p* = 0.03, $\eta_p^{2}$ = 0.16). Bonferroni post hoc tests revealed significant improvements from pre- to post- treatment on the SRS-2 for ASD participants (*p* = 0.008), which were maintained at follow-up (*p* > 0.05). TD teens had stable SRS-2 scores over time (*p*’s > 0.05), demonstrating significantly higher social responsiveness than ASD participants at each time point (all *p*’s < 0.001).

To examine parent-reported social engagement, a within-subjects three-way repeated measures ANOVA was conducted for ASD participants only. There was a significant main effect of time on hosted get-togethers for the ASD group (*F*(2, 24) = 8.84, *p* = 0.001, $\eta_p^{2}$ = 0.42). Bonferroni post hoc tests showed significant improvements from pre- to post- treatment (*p* < 0.01), which were maintained at follow-up (*p* > 0.05). A pre-post analysis conducted with TD adolescents (due to missing follow-up data) revealed stable scores on parent-reported hosted get-togethers between the first two timepoints (*p* > 0.05). Group differences between ASD and TD participants in the frequency of hosted get-togethers were significant at pre-treatment (*p* = 0.001), but were not significant at post-treatment (*p* = 0.67). For QSQ invited get-togethers, the group by time interaction was not significant, and scores were stable across time for both ASD and TD participants (all *p*’s > 0.05). Parents reported a similar frequency of invited get-togethers for ASD and TD participants across all time points (all *p*’s > 0.05). See Table 3 for detailed ANOVA results and scores at pre, post, and follow-up for ASD and TD participants.

**Table 3:** Group (2) by time (3) repeated measures ANOVA for ASD and TD participants

	Pre-intervention Mean (SD)	Post-intervention Mean (SD)	4-month follow-up Mean (SD)	*F*	*P*	Effect size (η_p_^2^)
*Parent-report measures*						
SRS total T-score^a^				4.22*	0.03	0.16
ASD	**74.85 (12.84)**	**68.85 (15.06)**	**68.69 (14.71)**			
TD	**44.00 (3.77)**	**42.55 (2.77)**	**46.00 (5.69)**			
SSIS social skills^a^				1.90	0.17	0.08
ASD	**81.62 (19.19)**	**87.85 (19.05)**	**90.31 (19.84)**			
TD	**106.64 (12.34)**	**107.09 (11.26)**	**108.09 (8.96)**			
SSIS prob behaviors^a^				0.89	0.40	0.04
ASD	**120.23 (17.74)**	**116.00 (18.51)**	**118.50 (18.02)**			
TD	**93.82 (7.92)**	**94.00 (10.11)**	**100.00 (16.89)**			
QSQ hosted^b^				8.84**	0.001	0.42
ASD	**0.23 (0.60)**	2.00 (1.47)	1.15 (1.46)			
TD	**2.18 (1.66)**	2.45 (3.42)	N/A			
QSQ invited^a^				0.001	0.99	0.00
ASD	0.69 (1.32)	1.08 (1.12)	1.15 (.99)			
TD	1.64 (1.43)	2.00 (1.55)	2.09 (1.45)			
*Adolescent Self-Report Measures*						
TASSK-R Total				38.72***	0.000	0.65
ASD	14.31 (2.62)	**25.00 (3.65)**	**24.00 (3.58)**			
TD	14.50 (3.17)	**15.80 (2.86)**	**15.10 (3.48)**			
LSDQ Total				0.53	0.60	0.02
ASD	**56.38 (13.07)**	**58.46 (10.95)**	**60.00 (11.37)**			
TD	**69.30 (10.02)**	**70.00 (10.37)**	**69.20 (5.20)**			
SIAS Total^a,b^				3.31	0.08	0.22
ASD	34.85 (18.10)	27.62 (14.63)	30.77 (15.93)			
TD	22.82 (12.81)	20.91 (14.70)	N/A			
PH2 Total				0.02	0.98	0.00
ASD	47.46 (11.00)	49.08 (5.60)	47.85 (10.28)			
TD	52.11 (10.11)	53.67 (10.82)	52.00 (9.91)			
FQS Total				2.16	0.13	0.11
ASD	**84.75 (12.91)**	86.62 (12.19)	86.69 (13.58)			
TD	**96.25 (10.01)**	93.00 (9.37)	91.63 (10.00)			
QSQ Hosted				0.02	0.98	0.00
ASD	**0.54 (.97)**	2.23 (2.52)	**1.15 (1.63)**			
TD	**2.80 (1.87)**	4.20 (5.77)	**3.30 (2.95)**			
QSQ Invited				0.20	0.82	0.01
ASD	**0.62 (1.19)**	1.00 (1.23)	0.77 (.93)			
TD	**2.20 (2.20)**	3.20 (3.99)	2.30 (2.95)			

*Note.* Group (2) by Time (3) Interaction, *p < 0.05; **p < 0.01; ***p <0 .001. Numbers in bold reflect between-group differences at the particular time-point

^a^The sphericity assumption was violated (i.e., Mauchley’s test was significant). The Greenhouse–Geisser adjustment was used to determine the F ratio, *p* value, and effect size estimates

^b^TD follow-up data was not collected for the SIAS and was missing for parent-reported QSQ-hosted get-togethers. Thus, a three-way within-group repeated measures ANOVA was conducted for the ASD group

### Corroboration of Findings from Observational Measure

Contextual Assessment of Social Skills (CASS) observational ratings were analyzed to examine whether perceived improvements in social functioning were corroborated by observed improvements in conversational skills. Though descriptive statistics revealed higher average ratings in several conversational domains from pre- to post- treatment, these improvements were not significant (all *p*’s > 0.05). Only two significant differences emerged from pre- to post-treatment: participants initiated significantly fewer topic changes (*p* < 0.05) and had greater kinesic arousal (i.e., demonstrated more fidgeting) at post-treatment (*p* < 0.05). See Table 4 for comparisons of selected CASS codes at pre- and post- intervention.

**Table 4:** Comparisons of selected CASS codes at pre- and post- intervention (*n* = 13)

	Pre-intervention	Post-intervention	*T*	*p*
	Mean (SD)	Mean (SD)		
# of Questions	3.77 (2.68)	2.77 (2.46)	1.93	.08
# of topic changes	2.69 (1.75)	1.92 (1.71)	2.25	0.04*
Vocal expressiveness	3.77 (1.83)	3.92 (2.10)	0.35	0.73
Gestures	3.00 (1.96)	3.92 (2.18)	1.45	0.17
Kinesic arousal	3.92 (1.61)	2.85 (1.41)	2.94	0.01*
Overall involvement	4.46 (1.51)	4.77 (1.69)	1.17	0.26
Overall rapport	4.23 (1.48)	4.23 (1.79)	0.00	1.00
CASS total score	15.15 (6.72)	13.69 (6.70)	−1.45	0.17

*Note.* Paired samples T-test, *p < .05. Vocal Expressiveness, Gestures, Kinesic Arousal, Overall Involvement, and Overall Rapport are scored on 1 to 7 scale; higher scores indicate better conversational skills. CASS Total Score = sum of # of questions asked, # of topic changes, overall involvement, and overall rapport

To examine whether observed improvements on the CASS were associated with perceived improvements in social functioning, bivariate Pearson correlations were conducted between the mean difference scores on the two global CASS items (overall involvement and rapport) and the CASS total score, and the SSIS, SRS-2, TASSK, and QSQ-hosted get-togethers. There was a significant correlation between change in CASS involvement and change on the SRS-2 (*r* = −0.56, *p* < 0.05), indicating that greater conversational involvement was associated with improved parent-reported social responsiveness. Additionally, there was a significant correlation between change in CASS involvement and change on the SSIS social skills standard score (*r* = 0.74, *p* < 0.01), indicating that greater conversational involvement was associated with improvement in parent-reported social skills. There was also a significant correlation between change in the CASS total score and change on the SSIS social skills standard score (*r* = 0.59, *p* < 0.05), indicating that greater overall conversational skills were associated with improved parent-reported social skills. Finally, there was a significant correlation between change in CASS quality of rapport and change in parent-reported hosted get-togethers on the QSQ (*r* = 0.60, *p* < 0.05), indicating that greater overall rapport was associated with an increase in hosted get-togethers. No other correlations were significant. See Table 5 for correlations between mean difference scores on the CASS global items and questionnaire measures from pre- to post- intervention.

**Table 5:** Correlations between mean difference scores on CASS global items and questionnaire measures from pre- to post- intervention (*n* = 13)

Variables	1	2	3	4	5	6	7
1. CASS Involvement	−						
2. CASS Rapport	0.36	−					
3. CASS Total	0.79**	0.54	−				
4. SRS-2 Total	−0.56*	−0.05	−0.32	−			
5. SSIS Social Skills	0.74**	0.26	0.59*	−0.83**	−		
6. SSIS Problem Behaviors	−0.17	0.41	0.15	0.66*	−0.32	−	
7. TASSK-R	0.09	−0.30	0.10	0.43	−0.36	0.11	−

*Note.* Bivariate Pearson correlations, *p < 0.05; **p < 0.01

## Discussion

The purpose of this pilot study was to examine perceived and observed changes in social functioning following an evidence-based social skills intervention with a predominantly Latinx sample. Consistent with previous PEERS studies (e.g., Laugeson et al., 2012; Rabin et al., 2018; Schohl et al., 2014; Shum et al., 2018; Yoo et al., 2014), preliminary findings with a small sample demonstrate that adolescents with ASD showed improvements in social skills, social responsiveness, social knowledge, and social engagement after intervention, which were maintained four months later. This is the first study to our knowledge to replicate these findings with a sample of predominantly Latinx participants and corroborate these findings using an observational measure of conversational skills and a control group of typically developing adolescents.

### Perceived Treatment Outcomes

The perceived improvements in adolescent social functioning in this study are consistent with the medium to large effects reported in previous studies (e.g., Rabin et al., 2018; Shum et al., 2018) evaluating the PEERS intervention (i.e., $\eta_p^{2}$ > 0.09 for medium effects, $\eta_p^{2}$ > 0.25 for large effects; Cohen, 1988; Miles & Shevlin, 2001). Despite the relatively small sample size in the current study, partial eta squared effect size estimates for the between-subjects three-way ANOVA ranged from 0.16 (for improved social responsiveness) to 0.65 (for increased social knowledge), reflecting medium to large effects. Furthermore, the maintenance of treatment gains in social responsiveness, social skills, social engagement, and social knowledge at four-month follow up were comparable to the results of similar studies, providing further validation for the durability of treatment outcomes in culturally diverse samples (e.g., Rabin et al., 2018; Shum et al., 2018; Yamada et al., 2020; Yoo et al., 2014).

One aim of this study was to evaluate how clinically meaningful the perceived improvements in social functioning were, by comparing ASD participants’ scores following treatment to a control group of TD participants. Results revealed that prior to treatment, participants with ASD had lower quality friendships and fewer get-togethers than their TD peers. After participating in PEERS, teens with ASD perceived their friendships to be similar in quality to that of TD teens. Furthermore, both adolescents with ASD and their parents reported a significant increase in the frequency of hosted get-togethers from pre- to post- treatment, such that the frequency of hosted get-togethers at post-treatment was similar to that of TD teens. Though there was a decrease in hosted get-togethers at the four-month follow-up, this was likely due to the assessment being conducted during the stay-at-home order in California during the COVID-19 pandemic. Nevertheless, after participating in PEERS, teens with ASD had similar levels of social engagement to those reported by TD participants and their parents. These findings suggest that participants with ASD had not only statistically significant improvements in social engagement and friendship quality, but also clinically meaningful, at least by conventional standards of normative instruments. However, despite improvements in parent-reported social responsiveness, social skills, and decreases in problem behaviors following treatment, participants with ASD were still rated by their parents as having more challenges in these areas than TD participants.

In addition, this study extends previous findings (e.g., Schohl et al., 2014; Yoo et al., 2014) related to a reduction in social anxiety following treatment. Though results were only marginally significant, adolescents with ASD reported lower average social anxiety from pre- to post- intervention, and overall had comparable scores to TD adolescents. This finding is contrary to what might be expected given the high rates of anxiety among adolescents with ASD (e.g., Bauminger & Kasari, 2000). This unintended effect may be a result of the intensive screening of participants prior to participation, in terms of social skills (rather than anxiety) being the primary treatment priority. Nevertheless, these findings are meaningful as the PEERS intervention does not specifically target anxiety; therefore, reductions in anxiety may be the result of greater confidence and comfort in social situations, perhaps due to learning and practicing social skills. It is noteworthy that there were no significant improvements in adolescent perceived loneliness or self-concept in this study. It is possible that these outcomes reflect the dilemma of social skills interventions being based on a neurotypical stance. Alternatively, it may be that teens require additional time to use the newly learned skills to form friendships, and improvements in these outcomes may not be seen until more months elapse following intervention.

### Observed Treatment Outcomes

The third aim of this study was to examine whether perceived treatment gains were corroborated by observed gains in conversational skills with an unfamiliar peer (CASS; Ratto et al., 2011). The CASS total score (comprised of question asking, topic changes, conversational involvement, and overall rapport) did not change from pre- to post- intervention, unlike in the study by Rabin et al. (2018), who found a significant increase in the average CASS total score. However, the CASS total score may have limited utility in evaluating treatment gains following PEERS, as the appropriate frequency of question asking and topic changes varies depending on the individual. For example, an increase in the number of questions asked may reflect an improvement for some, while a decrease may reflect a positive change for others, as their baseline levels of question asking may have been inappropriately high. Due to the tremendous variability in the characteristics and ability levels in individuals with ASD, evaluating baseline social needs and treatment effects at an individual level is critical for assessing the effectiveness of an intervention (Lord et al., 2005).

One other possibility is that the CASS items do not capture the specific skills that would be expected to change following PEERS. Thus, adding additional items to the CASS paradigm may be necessary to better reflect treatment-related improvements in conversational skills (Van Pelt et al., 2020). Overall, these findings highlight the complexity of assessing treatment outcomes following social skills intervention, and the need for further development and evaluation of observational measures of treatment outcome.

### Relationship Between Observed and Perceived Treatment Outcomes

To validate the treatment gains perceived by adolescents and parents participating in PEERS, we examined the relationship between observed improvements in conversational skills and parent and self- reported improvements in social competence. Improvements in the global domain of conversational involvement on the CASS were related to improvements in parent-reported social responsiveness on the SRS-2 and social skills on the SSIS. Though Dolan et al. (2016) found associations between improvements in observed overall rapport and adolescent-reported social skills knowledge, the associations in the current study were between observed rapport and parent-reported social engagement. Thus, the current findings reflect a positive relationship between practicing social skills during get-togethers and implementing these skills with an unfamiliar person. Using the observational measure of conversational skills also eliminates some of the shared method variance imposed by self- and parent-reported gains on questionnaires.

## Limitations and Future Directions

The obvious first limitation is the small sample size, which affects the number and significance of analyses. There are a few other notable limitations. First, participants in this study were not randomized to a treatment or waitlist control group. Rather, this study used a convenience sample to assess the preliminary effectiveness of the PEERS social skills intervention with primarily Latinx families, which may have led to a potential selection bias (i.e., only highly motivated families enrolled). Future research should incorporate a randomized approach to validate the PEERS intervention with Latinx and diverse populations.

Second, for the purpose of ease and efficiency, the research confederates used in this study for the CASS were college students and were therefore not same-age peers. Participants may have perceived them as “adults” rather than as peers. Future research should recruit students closer in age to the participants to better gauge how adolescents interact with same-age peers. Third, this study only includes pre- and post- data on the CASS, as in-person follow-up appointments were not possible for the second cohort due to the COVID-19 pandemic. Future studies should include follow-up data using the CASS to evaluate the maintenance of observed treatment gains after adolescents have had several months to practice the newly learned skills. Fourth, as previous studies have found significant differences on the CASS between TD and ASD participants without treatment (e.g., Ratto et al., 2011), future PEERS studies should consider administering the CASS to a control group of TD participants at pre- and post- intervention to examine whether these differences decrease with treatment.

Finally, as the purpose of PEERS is to develop close meaningful friendships with same-age peers, the concepts taught in the program go above and beyond conversational skills. For example, PEERS teaches the skills for handling arguments with friends, responding to teasing and bullying, and planning and organizing get-togethers with friends. Thus, observing how participants interact with familiar peers at school or at their extracurricular activities may reflect a more accurate representation of treatment gains.

In conclusion, this study extends previous findings on the perceived and observed effects of the PEERS intervention, and provides preliminary evidence for the successful delivery of PEERS in both English and Spanish. Findings suggest that adolescents and their parents who participated in PEERS perceived positive changes in social functioning, which were somewhat corroborated by observed changes in conversational skills.

## Footnotes

1 Due to a lack of universally accepted terminology for describing autism (e.g., Kenny et al., 2016), this paper alternates between using person-first (i.e., person with autism) and identity-first (i.e., autistic person) language.

## References

[CR1] Asher S, Hymel S, Renshaw P (1984). Loneliness in children. Child Development.

[CR2] Baron-Cohen S, Richler J, Bisarya D, Gurunathan N, Wheelwright S (2003). The systemizing quotient: An investigation of adults with Asperger syndrome or high–functioning autism, and normal sex differences Philosophical Transactions of the Royal Society of London. Series b: Biological Sciences.

[CR3] Bauminger N, Kasari C (2000). Loneliness and friendship in high-functioning children with autism. Child Development.

[CR4] Bauminger N, Shulman C, Agam G (2003). Peer interaction and loneliness in high functioning children with autism. Journal of Autism and Developmental Disorders.

[CR5] Bernal G, Domenech Rodríguez MM (2009). Advances in Latino family research: Cultural adaptations of evidence-based interventions. Family Process.

[CR6] Bukowski WM, Hoza B, Boivin M (1994). Measuring friendship quality during pre-and early adolescence: The development and psychometric properties of the Friendship Qualities Scale. Journal of Social and Personal Relationships.

[CR7] Carter AS, Davis NO, Klin A, Volkmar FR, Volkmar FR, Paul R, Klin A, Cohen D (2005). Social development in autism. Handbook of autism and pervasive developmental disorders.

[CR8] Chevallier C, Kohls G, Troiani V, Brodkin ES, Schultz RT (2012). The social motivation theory of autism. Trends in Cognitive Sciences.

[CR9] Chlebowski C, Hurwich-Reiss E, Wright B, Brookman-Frazee L (2020). Using stakeholder perspectives to guide systematic adaptation of an autism mental health intervention for Latinx families: A qualitative study. Journal of Community Psychology.

[CR10] Church C, Alisanski S, Amanullah S (2000). The social, behavioral, and academic experiences of children with Asperger syndrome. Focus on Autism and Other Developmental Disabilities.

[CR11] Cohen, J. (1988). *Statistical analysis for the behavioral sciences.* Hillsdale: Lawrance Erlbaum.

[CR12] Constantino, J., & Gruber, C. (2012). *Social Responsiveness Scale – Second Edition (SRS-2).* Western Psychological Services: Torrance, CA.

[CR13] Dolan BK, Van Hecke AV, Carson AM, Karst JS, Stevens S, Schohl KA, Hummel E (2016). Brief report: Assessment of intervention effects on in vivo peer interactions in adolescents with autism spectrum disorder (ASD). Journal of Autism and Developmental Disorders.

[CR14] Ellingsen, R., Bolton, C., & Laugeson, E. (2017). Evidence-based social skills groups for individuals with autism spectrum disorder across the lifespan. In J. Leaf (Ed.), *Handbook of Social Skills and Autism Spectrum Disorder* (pp. 343–358). New York: Springer. 10.1007/978-3-319-62995-7_20

[CR15] Gates JA, Kang E, Lerner MD (2017). Efficacy of group social skills interventions for youth with autism spectrum disorder: A systematic review and meta-analysis. Clinical Psychology Review.

[CR16] Gresham F, Elliott SN (2007). Social Skills Improvement System (SSIS) Rating Scales.

[CR17] Happé F, Frith U (2006). The weak coherence account: Detail-focused cognitive style in autism spectrum disorders. Journal of Autism and Developmental Disorders.

[CR18] Huey SJ, Polo AJ (2008). Evidence-based psychosocial treatments for ethnic minority youth. Journal of Clinical Child & Adolescent Psychology.

[CR19] Jurgens, A. (2020). Neurodiversity in a neurotypical world: An enactive framework for investigating autism and social institutions. In H. Bertilsdotter-Rosqvist, N. Chown, & A. Stenning (Eds.), *Neurodiversity studies: A new critical paradigm*, (pp. 73–88). London: Routledge. Kaat, A. J., & Lecavalier, L. (2014). Group-based social skills treatment: A methodological review. *Research in Autism Spectrum Disorders, 8*(1), 15–24. 10.1016/j.rasd.2013.10.007

[CR20] Kenny L, Hattersley C, Molins B, Buckley C, Povey C, Pellicano E (2016). Which terms should be used to describe autism? Perspectives from the UK autism community. Autism.

[CR21] Knott F, Dunlop AW, Mackay T (2006). Living with ASD: How do children and their parents assess their difficulties with social interaction and understanding?. Autism.

[CR22] Laugeson EA (2014). The PEERS curriculum for school based professionals: Social skills training for adolescents with autism spectrum disorder.

[CR23] Laugeson EA, Frankel F (2010). Social skills for teenagers with developmental and autism spectrum disorder: The PEERS treatment manual.

[CR24] Laugeson EA, Frankel F, Gantman A, Dillon AR, Mogil C (2012). Evidence-based social skills training for adolescents with autism spectrum disorders: The UCLA PEERS program. Journal of Autism and Developmental Disorders.

[CR25] Laugeson EA, Frankel F, Mogil C, Dillon AR (2009). Parent-assisted social skills training to improve friendships in teens with autism spectrum disorders. Journal of Autism and Developmental Disorders.

[CR26] Locke J, Ishijima EH, Kasari C, London N (2010). Loneliness, friendship quality and the social networks of adolescents with high-functioning autism in an inclusive school setting. Journal of Research in Special Educational Needs.

[CR27] Lord C, Rutter M, DiLavore PC, Risi S, Gotham K, Bishop SL (2012). Autism Diagnostic Observation Schedule.

[CR28] Lord C, Wagner A, Rogers S, Szatmari P, Aman M, Charman T, Harris S (2005). Challenges in evaluating psychosocial interventions for autistic spectrum disorders. Journal of Autism and Developmental Disorders.

[CR29] Mash EJ, Hunsley J (2005). Evidence-based assessment of child and adolescent disorders: Issues and challenges. Journal of Clinical Child and Adolescent Psychology.

[CR30] Mattick RP, Clarke JC (1998). Development and validation of measures of social phobia scrutiny fear and social interaction anxiety. Behaviour Research and Therapy.

[CR31] Miles J, Shevlin M (2001). Applying regression and correlation: A guide for students and researchers.

[CR32] Pickard K, Reyes N, Reaven J (2019). Examining the inclusion of diverse participants in cognitive behavior therapy research for youth with autism spectrum disorder and anxiety. Autism.

[CR33] Piers, E. V. (1984). *Piers-Harris Children's Self-Concept Scale*. Los Angeles: Western Psychological Services.

[CR34] Rabin SJ, Israel-Yaacov S, Laugeson EA, Mor-Snir I, Golan O (2018). A randomized controlled trial evaluating the Hebrew adaptation of the PEERS^®^ intervention: Behavioral and questionnaire-based outcomes. Autism Research.

[CR35] Ratto AB, Anthony BJ, Pugliese C, Mendez R, Safer-Lichtenstein J, Dudley KM, Anthony LG (2017). Lessons learned: Engaging culturally diverse families in neurodevelopmental disorders intervention research. Autism.

[CR36] Ratto AB, Turner-Brown L, Rupp BM, Mesibov GB, Penn DL (2011). Development of the contextual assessment of social skills (CASS): A role play measure of social skill for individuals with high-functioning autism. Journal of Autism and Developmental Disorders.

[CR37] Reichow B, Volkmar FR (2010). Social skills interventions for individuals with autism: Evaluation for evidence-based practices within a best evidence synthesis framework. Journal of Autism and Developmental Disorders.

[CR38] Schohl KA, Van Hecke AV, Carson AM, Dolan B, Karst J, Stevens S (2014). A replication and extension of the PEERS intervention: Examining effects on social skills and social anxiety in adolescents with autism spectrum disorders. Journal of Autism and Developmental Disorders.

[CR39] Shattuck PT, Orsmond GI, Wagner M, Cooper BP (2011). Participation in social activities among adolescents with an autism spectrum disorder. PLoS ONE.

[CR40] Shum KKM, Cho WK, Lam LMO, Laugeson EA, Wong WS, Law LS (2018). Learning how to make friends for Chinese adolescents with autism spectrum disorder: A randomized controlled trial of the Hong Kong Chinese version of the PEERS® intervention. Journal of Autism and Developmental Disorders.

[CR41] Simmons GL, Ioannou S, Smith JV, Corbett BA, Lerner MD, White SW (2020). Utility of an observational social skill assessment as a measure of social cognition in autism. Autism Research.

[CR42] Stratis EA, Lecavalier L (2015). Informant agreement for youth with autism spectrum disorder or intellectual disability: A meta-analysis. Journal of Autism and Developmental Disorders.

[CR43] Van Pelt BJ, Idris S, Jagersma G, Duvekot J, Maras A, Van Der Ende J, Van Haren NEM, Greaves-Lord K (2020). The ACCEPT-study: Design of an RCT with an active treatment control condition to study the effectiveness of the Dutch version of PEERS^®^ for adolescents with autism spectrum disorder. BMC Psychiatry.

[CR44] Wechsler, D. (2011). *Wechsler Abbreviated Scale of Intelligence–Second Edition (WASI-II).* San Antonio, TX: NCS Pearson.

[CR45] White SW, Scarpa A, Conner CM, Maddox BB, Bonete S (2015). Evaluating change in social skills in high-functioning adults with autism spectrum disorder using a laboratory-based observational measure. Focus on Autism and Other Developmental Disabilities.

[CR46] Wolstencroft J, Robinson L, Srinivasan R, Kerry E, Mandy W, Skuse D (2018). A systematic review of group social skills interventions, and meta-analysis of outcomes, for children with high functioning ASD. Journal of Autism and Developmental Disorders.

[CR47] Yamada T, Miura Y, Oi M, Akatsuka N, Tanaka K, Tsukidate N, Laugeson EA (2020). Examining the treatment efficacy of PEERS in Japan: Improving social skills among adolescents with Autism Spectrum Disorder. Journal of Autism and Developmental Disorders.

[CR48] Yoo HJ, Bahn G, Cho IH, Kim EK, Kim JH, Min JW, Cho S (2014). A randomized controlled trial of the Korean version of the PEERS® parent-assisted social skills training program for teens with ASD. Autism Research.

[CR49] Zeedyk SM, Rodriguez G, Tipton LA, Baker BL, Blacher J (2014). Bullying of youth with autism spectrum disorder, intellectual disability, or typical development: Victim and parent perspectives. Research in Autism Spectrum Disorders.

[CR50] Zheng S, Kim H, Salzman E, Ankenman K, Bent S (2021). Improving social knowledge and skills among adolescents with autism: Systematic review and meta-analysis of UCLA PEERS® for Adolescents. Journal of Autism and Developmental Disorders.

